# A Simple Attitude Control of Quadrotor Helicopter Based on Ziegler-Nichols Rules for Tuning PD Parameters

**DOI:** 10.1155/2014/280180

**Published:** 2014-12-29

**Authors:** ZeFang He, Long Zhao

**Affiliations:** ^1^Science and Technology on Aircraft Control Laboratory, Beihang University, Beijing 100191, China; ^2^Digital Navigation Center, Beihang University, Beijing 100191, China

## Abstract

An attitude control strategy based on Ziegler-Nichols rules for tuning PD (proportional-derivative) parameters of quadrotor helicopters is presented to solve the problem that quadrotor tends to be instable. This problem is caused by the narrow definition domain of attitude angles of quadrotor helicopters. The proposed controller is nonlinear and consists of a linear part and a nonlinear part. The linear part is a PD controller with PD parameters tuned by Ziegler-Nichols rules and acts on the quadrotor decoupled linear system after feedback linearization; the nonlinear part is a feedback linearization item which converts a nonlinear system into a linear system. It can be seen from the simulation results that the attitude controller proposed in this paper is highly robust, and its control effect is better than the other two nonlinear controllers. The nonlinear parts of the other two nonlinear controllers are the same as the attitude controller proposed in this paper. The linear part involves a PID (proportional-integral-derivative) controller with the PID controller parameters tuned by Ziegler-Nichols rules and a PD controller with the PD controller parameters tuned by GA (genetic algorithms). Moreover, this attitude controller is simple and easy to implement.

## 1. Introduction

Unmanned helicopters have been widely applied in military and commercial fields [1, 2]. Unmanned four-rotor helicopters (quadrotor helicopters) are mostly used for search and rescue, surveillance, reconnaissance, data acquisition, and so forth. Their potential applications include border patrol, widefire monitoring, traffic monitoring, mineral exploration, and transportation [3–5]. Compared to conventional helicopters, quadrotor helicopters show many advantages. Quadrotor helicopters have hovering and VTOL (vertical takeoff and landing) capabilities which are also the characteristic of conventional helicopters. In other words, they have large maneuverability, do not require large takeoff and landing site, and can execute special tasks in dangerous and inaccessible environments. Furthermore, conventional helicopters are structurally complex, expensive, and hard to control, while quadrotor helicopters are mechanically simple, demand low manufacturing and operational costs, and are easy to control. Therefore, quadrotor helicopters draw intensive attention, and relevant technologies have also been an important research topic in recent years [6]. Quadrotor helicopter control is a challenging problem because a quadrotor helicopter is a complex system with high nonlinearities, strong couplings, and underactuation [7]. Therefore, the research for the control system of quadrotor helicopters has been widely conducted.

The methods for establishing a nonlinear model of quadrotor helicopters include white box modeling, black box modeling, and grey box modeling. Grey box modeling is commonly used in quadrotor modeling papers. The procedure for modeling quadrotor by the grey box modeling method is as follows: firstly, system model is written with the method of mechanism modeling; secondly, because some of the parameters in the model are unknown, they need to be derived by identification methods. There are two main mechanism modeling methods: Newton-Euler formalism and Lagrange-Euler formalism. In [8], a nonlinear model was proposed by representing the quadrotor helicopters kinematics and dynamics characteristics based on Newton-Euler formalism. In [9], the same methodology was used to obtain the motion equations and the rotor dynamics were considered as well during the model establishment. The authors who established the quadrotor helicopters model using the Newton-Euler formalism in [9] described the quadrotor helicopters' dynamics by the Lagrange-Euler formalism in [10]. In this paper, quadrotor helicopter motion equations were obtained by the Newton-Euler formalism without considering the rotor dynamics during the model establishment.

The system equations were rewritten in state space for the controller design. The quadrotor motion can be split into two motions: the angular rotation motion and the linear translation motion. From the system equations, it can be seen that the rotational motion is independent of the translational motion, while the translational motion depends on the rotational motion. The quadrotor control structure is usually divided into internal and external loops according to the characteristics of the system model. The former is used to control attitude and the latter to control position. According to the control strategy which internal and external loop controls take, the quadrotor control can be divided into inner-outer loop control and full control. In inner-outer loop control methods, internal and external loops use different control strategies while, in full control methods, the same control strategy is used by internal and external loops. Different control methods which were recently tested on quadrotor helicopters are as follows: classic PID control [11], all kinds of improved methods of PID [6, 12], *H*_*∞*_ control [4], predictive control [4], backstepping control [5, 7, 9], sliding mode control [9], LQR control [10], dynamic surface control [13], adaptive control [14, 15], neural network control [16], fuzzy control [17, 18], and the integrated method combined each other's advantages such as integral sliding mode control [3], integral backstepping control [19], fuzzy integral sliding mode control based on backstepping [20], and robust adaptive control [21–23]. Despite that the development of control methods has experienced the classical control, modern control, and intelligent control which largely improve the control effect, the classic PID control is still the most common and practical one in engineering controller design [24] because of its simplicity of control principle. An important step in the PID controller design is to tune PID controller parameters. Engineers generally use empirical methods to derive adjustable parameter, but it is time-consuming and thus inefficient. As such, a variety of intelligent PID parameter tuning methods have been proposed and studied in the literature. However, as with intelligent control methods, the parameter tuning methods are too complicated to be mastered by engineering technicians despite the good effect. The Ziegler-Nichols rules for tuning the PID controller, which were proposed by Ziegler and Nichols in 1942, have been widely recognized and used as described in [25] because it is simple and practical. In addition, the feedback linearization method [26] is also a very good method to decouple and linearize quadrotor models. In view of the advantages of the Ziegler-Nichols rules for tuning the PID controller and the feedback linearization method, the quadrotor attitude control strategy adopted in this paper is depicted as follows. The PD controller tuned by Ziegler-Nichols rules is firstly applied to the linearized model of quadrotor after the feedback linearization. Further, the nonlinear controller consisting of the PD control and feedback linearization item is applied to the quadrotor controlled object. The PD controller with PD parameters tuned by Ziegler-Nichols rules is first applied to quadrotor Helicopter in this paper.

The remainder of the paper is organized as follows: the quadrotor helicopter model is described in Section 2. The control strategy is proposed in Section 3. In Section 4, the controller design principle is presented and contains feedback linearization, Ziegler-Nichols ruler for adjusting parameters, the controller parameter choice, and final form. Simulation results are reported in Section 5. Finally, the major conclusions of the paper are exposed in Section 6.

## 2. Quadrotor Model

**Figure 1:**
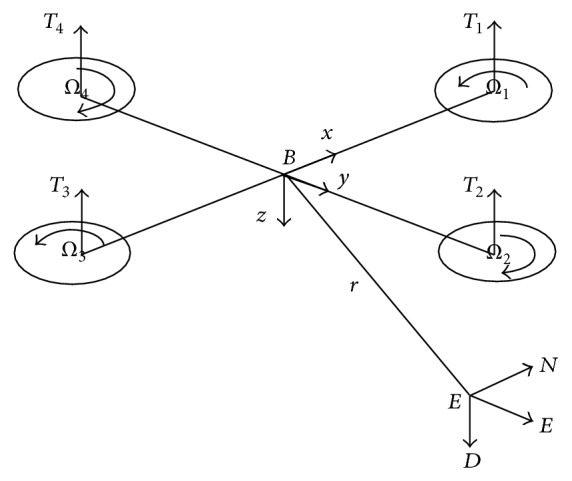
The structure of a quadrotor helicopter.

A quadrotor helicopter uses four rotors as the direct power of flight. Four rotors with the same structure and radius are symmetrically located on the four edges of a cross formed by two arms and located on a plane at the same height. Rotor 1 and rotor 3 rotate in the counterclockwise direction and rotor 2 and rotor 4 rotate in the clockwise direction. Four motors were symmetrically installed on the stent ends of the quadrotor; flight control computer and external equipment were installed in the space of the stent middle. The structure of a quadrotor helicopter is shown in Figure 1, where *B* represents the body coordinate system and *G* represents the ground coordinate system.

The attitude and location of a quadrotor helicopter can be controlled to desired values by changing four motor speeds (which results in the changes of four rotor speeds and corresponding lift). The following several kinds of force and moment can be performed on the quadrotor helicopter: the lifts caused by rotors rotation, the pitching moment and rolling moment caused by the difference of four rotors lift, the gravity, the gyroscopic effect, and aerodynamic torque effect, that is, yawing moment. The gyroscopic effect only appears in the lightweight construction quadrotor helicopter. Yawing moment is caused by unbalanced four rotors rotational speeds. The gyroscopic effect and yawing moment are in essence caused by the reaction torques. The reaction torques are caused by the rotor rotation; its direction is exactly opposite of the rotor rotation direction. Yaw moment can be cancelled when two rotors rotate in the opposite direction. The gyroscopic effect and aerodynamic torque effect are offset when the quadrotor hovers.

The space motion of the rigid body aircraft can be divided into two parts: the centre of mass movement and movement around the centre of mass. Six degrees of freedom are required in describing any time space motion. They are three barycenter movements and three angular motions, namely, three translation and three rotation motions along three axes. The control for six degrees of freedom motions can be implemented by adjusting the rotational speeds of different motors. Basic motions include forward and backward movements, lateral movement, vertical motion, roll motion, and pitch and yaw motions. The yaw motion of the quadrotor helicopter can be realised by a reactive torque produced by the rotor. The size of the reactive torque is relative to the rotor speed. When the four rotor speeds are the same, the reactive torques will offset by each other and quadrotor will remain still, whereas if the four rotor speeds are not absolutely same, the reactive torques will not be absolutely offset, and the quadrotor will start to rotate. The rotation of the quadrotor should be eliminated during the other motions of the quadrotor to ensure the stability of motions. That the four rotor speeds synchronously increase and decrease is also required in the vertical movement. When the quadrotor helicopter flies in the pitch and roll motion mode, horizontal motion along *x*- and *y*-axes will be triggered. In order to obtain the subduction motion and forward movement, the speed of the rear rotor must be increased and the speed of the front rotor must be decreased; on the contrary, nose-up pitch movement and backward movement can be produced. Similarly, the roll movement and lateral movement can be obtained by controlling the left rotor and right rotor.

Because of four inputs and six state outputs in a quadrotor, the quadrotor is considered an underactuated complex system. In order to control it, some assumptions are made in the process of quadrotor modeling as follows: quadrotor is a rigid body; the structure is symmetric; the centre of gravity and the origin of body coordinate system are coincident and ground effect is ignored.

The causes of the lift, gravity, and aerodynamic torque acting on a quadrotor differ from each other. Thus, it is very important to choose the appropriate coordinate system to exactly describe the quadrotor space motion state. For example, it is more convenient to describe the gravity of the aircraft in the ground coordinate frame and to describe the lift acting on the quadrotor; the body coordinate frame is more suitable. Therefore, when establishing a quadrotor motion model, reasonably choosing different coordinate frames is one of the important links to define and describe various motion parameters of the quadrotor.

Given an aircraft, the positive *x* direction in the body coordinate frame is usually defined as the moving direction, the positive *y* direction as the right side of the moving direction, and the positive *z* direction as the vertical downward direction; this is also termed forward-right-downward coordinate frame [26].

Because the fly altitude of quadrotor helicopters is limited in the atmosphere, the “flat earth hypothesis” can be adopted to consider the ground coordinate frame as an inertial coordinate frame to simplify the modeling complexity. In order to facilitate navigation and way-point tracking, the axis directions of the ground coordinate frame are chosen as north-east-down navigation frame directions, namely, that the *X*_*g*_ axis points to the north, the *Y*_*g*_ axis points to the east, and the *Z*_*g*_ axis is perpendicular to the ground and points to the center of the earth.

The procedure of the Newton-Euler formalism modeling is to project lift forces acting on the aircraft to the ground coordinate frame and analyze the linear motion of the aircraft with Newton's second law in the inertial coordinate system and the angular motion of the aircraft with the law of moment of momentum in the body coordinate system.

### 2.1. Kinematics Model

The transformation matrix between two rectangular coordinate systems is orthogonal. *R*(*x*, *ϕ*), *R*(*y*, *θ*), and *R*(*z*, *ψ*) denote rotation matrices produced by the ground coordinate frame rotating roll angle *ϕ*, pitch angle *θ*, and yaw angle *ψ* around *x*-, *y*-, and *z*-axes, respectively, and the expressions are as follows:

(1) Rx,ϕ=1000cos⁡⁡ϕsin⁡ϕ0−sin⁡ϕcos⁡⁡ϕ,Ry,θ=cos⁡⁡θ0−sin⁡θ010sin⁡θ0cos⁡⁡θ,Rz,ψ=cos⁡⁡ψsin⁡ψ0−sin⁡ψcos⁡⁡ψ0001.

The rotation matrix from the ground coordinate system to the body coordinate system is the product of formulae (1), which denote rotation around the *z*-axis followed by rotation around *y*-axis and finally followed by rotation around *x*-axis; namely,

(2) $$\begin{array}{c} R_{E\to B}=R\left(x,\phi \right)R\left(y,\theta \right)R\left(z,\psi \right). \end{array}$$

Therefore, the transformation matrix from the ground coordinate system to the body coordinate system is given by

(3) $$\begin{array}{c} R_{B\to E}=R_{E\to B}^{T}. \end{array}$$

The specific expression is given by

(4) $$\begin{array}{c} R_{B\to E}=\left[\begin{array}{ccc} c_{\theta }c_{\psi } & s_{\phi }s_{\theta }c_{\psi }-c_{\phi }s_{\psi } & c_{\phi }s_{\theta }c_{\psi }+s_{\phi }s_{\psi }\\ c_{\theta }s_{\psi } & s_{\phi }s_{\theta }s_{\psi }+c_{\phi }c_{\psi } & c_{\phi }s_{\theta }s_{\psi }-s_{\phi }c_{\psi }\\ -s_{\theta } & s_{\phi }c_{\theta } & c_{\phi }c_{\theta } \end{array}\right], \end{array}$$

where *c*_·_ = cos⁡⁡(·) and *s*_·_ = sin⁡(·).

**Figure 2:**
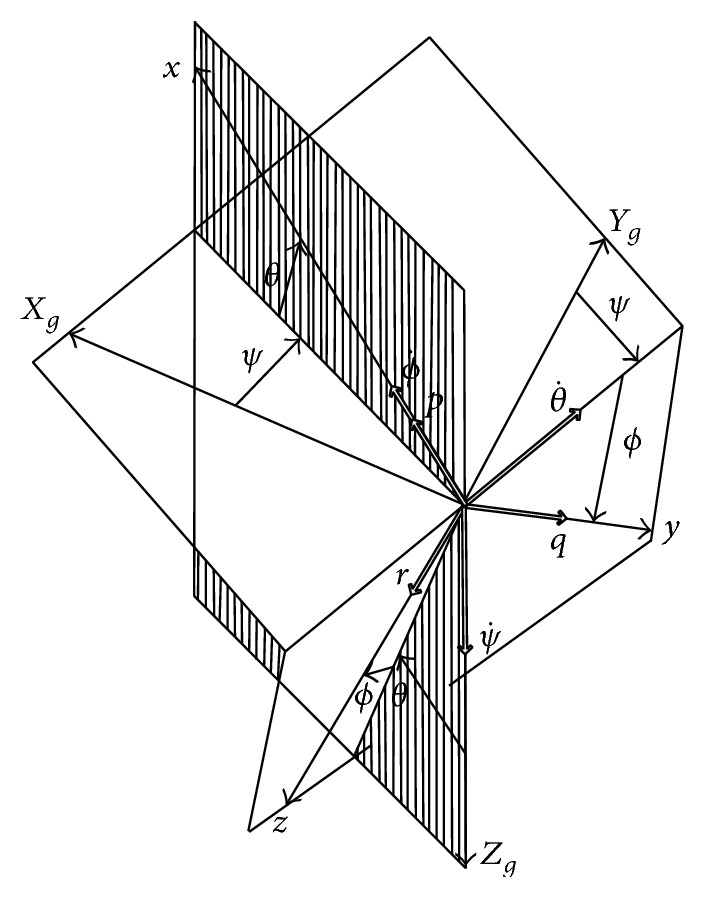
The relationships between angular velocity components and the attitude angle change rate.

The angular velocity components *p*, *q*, and *r* are the projection values on the body coordinate system of rotation angular velocity *ω* which denotes the rotation from the ground coordinate system to the body coordinate system. The relationships between angular velocity components and the attitude angle change rates are shown in Figure 2.

The transformation matrix from ${\left[\dot{\phi }\;\dot{\theta }\;\dot{\psi }\right]}^{T}$ to [*p* *q* *r*]^*T*^ is given by

(5) $$\begin{array}{c} \left[\begin{array}{c} p\\ q\\ r \end{array}\right]=R_{r}\left[\begin{array}{c} \dot{\phi }\\ \dot{\theta }\\ \dot{\psi } \end{array}\right], \end{array}$$

where

(6) $$\begin{array}{c} R_{r}=\left[\begin{array}{ccc} 1 & 0 & -\sin\theta \\ 0 & \cos\phi & \sin\phi \cos\theta \\ 0 & -\sin\phi & \cos\phi \cos\theta \end{array}\right]. \end{array}$$

Around hovering position, *R*_*r*_ is assumed as a unit matrix [26].

### 2.2. Dynamic Model

The dynamics model is composed of the rotational and translational motions. The rotational motion is fully actuated, while the translational motion is underactuated. In the body coordinate system, the rotational motion equations are derived according to the law of momentum theorem and gyroscopic effect of quadrotor, and they are given by

(7) $$\begin{array}{c} J\dot{\omega }+\omega \times J\omega +\omega \times \left[\begin{array}{ccc} 0 & 0 & J_{r}\Omega_{r} \end{array}\right]=M_{B}, \end{array}$$

where *J* is the inertia matrix of quadrotor which is diagonal under the hypothesis of structure symmetry and the elements *I*_*x*_, *I*_*y*_, and *I*_*z*_ are, respectively, inertia matrices of *x*-, *y*-, and *z*-axes. The last item on the left side of (7) represents the gyroscopic effect which is caused by the inertia of the rotors *J*_*r*_ and relative speed *Ω*_*r*_ = −*Ω*_1_ + *Ω*_2_ − *Ω*_3_ + *Ω*_4_, where *Ω*_*i*_  (*i* = 1,2, 3,4) represents the *i*th rotor speed. The aerodynamic force and moment produced by the *i*th rotor are directly proportional to the square of the rotor speed. The relationships are given by

(8) Fi=bΩi2,Mi=dΩi2,

where *b* and *d* are the aerodynamic force and moment constants, respectively. The moments acting on the quadrotor in the body coordinate system are given by

(9) $$\begin{array}{c} M_{B}=\left[\begin{array}{c} l\cdot b\left(-\Omega_{2}^{2}+\Omega_{4}^{2}\right)\\ l\cdot b\left(\Omega_{1}^{2}-\Omega_{3}^{2}\right)\\ d\left(\Omega_{1}^{2}-\Omega_{2}^{2}+\Omega_{3}^{2}-\Omega_{4}^{2}\right) \end{array}\right], \end{array}$$

where *l* is the moment arm which represents the distance from the axis of a rotor to the center of quadrotor.

The translational motion equations are obtained in the ground coordinate system by the method of Newton's second law:

(10) $$\begin{array}{c} m\ddot{r}={\left[\begin{array}{ccc} 0 & 0 & mg \end{array}\right]}^{T}+RF_{B}, \end{array}$$

where *r* = [*x* *y* *z*]^*T*^ is the position of quadrotor in the ground coordinate system, *m* is the mass of quadrotor, *g* is the acceleration of gravity, and *F*_*B*_ is the total lift force acting on quadrotor in the body coordinate system; namely,

(11) $$\begin{array}{c} F_{B}=\left[\begin{array}{c} 0\\ 0\\ -b\left(\Omega_{1}^{2}+\Omega_{2}^{2}+\Omega_{3}^{2}+\Omega_{4}^{2}\right) \end{array}\right]. \end{array}$$

### 2.3. The Motion Equations of Quadrotor

Synthesizing the kinematics and dynamics models of quadrotor, the motion equations of quadrotor can be derived as follows:

(12) ϕ¨=θ˙ψ˙Iy−IzIx−JrIxθ˙Ωr+LIxU2,θ¨=ϕ˙ψ˙Iz−IxIy+JrIyϕ˙Ωr+LIyU3,ψ¨=ϕ˙θ˙Ix−IyIz+1IzU4,x¨=−U1mcos⁡⁡ϕsin⁡θcos⁡⁡ψ+sin⁡ϕsin⁡ψ,y¨=−U1mcos⁡⁡ϕsin⁡θsin⁡ψ−sin⁡ϕcos⁡⁡ψ,z¨=g−U1mcos⁡⁡ϕcos⁡⁡θ,

where *U*_1_, *U*_2_, *U*_3_, and *U*_4_ are the control input variables, which can be calculated by *U*_1_ = *b*(*Ω*_1_^2^ + *Ω*_2_^2^ + *Ω*_3_^2^ + *Ω*_4_^2^), *U*_2_ = *b*(−*Ω*_2_^2^ + *Ω*_4_^2^), *U*_3_ = *b*(*Ω*_1_^2^ − *Ω*_3_^2^), and *U*_4_ = *d*(*Ω*_1_^2^ − *Ω*_2_^2^ + *Ω*_3_^2^ − *Ω*_4_^2^), respectively.

## 3. Control Strategy

The state space model adopted by the control system is $\dot{X}=f\left(X,U\right)$, where *X* is the state vector and *U* is the control input vector. The state vector is chosen as $X={\left[x\;y\;z\;\dot{x}\;\dot{y}\;\dot{z}\;\phi \;\theta \;\psi \;\dot{\phi }\;\dot{\theta }\;\dot{\psi }\right]}^{T}$. In the design of controller, the state variables are chosen as *x*_1_ = *x*, *x*_2_ = *y*, *x*_3_ = *z*, $x_{4}={\dot{x}}_{1}=\dot{x}$, $x_{5}={\dot{x}}_{2}=\dot{y}$, $x_{6}={\dot{x}}_{3}=\dot{z}$, *x*_7_ = *ϕ*, *x*_8_ = *θ*, *x*_9_ = *ψ*, $x_{10}={\dot{x}}_{7}=\dot{\phi }$, $x_{11}={\dot{x}}_{8}=\dot{\theta }$, and $x_{12}={\dot{x}}_{9}=\dot{\psi }$.

Synthesizing the motion equations of quadrotor, the state vector, and the control input variables, the state equations can be described as

(13) $$\begin{array}{c} f\left(X,U\right)=\left[\begin{array}{c} x_{4}\\ x_{5}\\ x_{6}\\ -\frac{U_{1}}{m}\left(\cos x_{7}\sin x_{8}\cos x_{9}+\sin x_{7}\sin x_{9}\right)\\ -\frac{U_{1}}{m}\left(\cos x_{7}\sin x_{8}\sin x_{9}-\sin x_{7}\cos x_{9}\right)\\ g-\frac{U_{1}}{m}\left(\cos x_{7}\cos x_{8}\right)\\ x_{10}\\ x_{11}\\ x_{12}\\ x_{11}x_{12}\frac{I_{y}-I_{z}}{I_{x}}-\frac{J_{r}}{I_{x}}x_{11}\Omega_{r}+\frac{L}{I_{x}}U_{2}\\ x_{10}x_{12}\frac{I_{z}-I_{x}}{I_{y}}+\frac{J_{r}}{I_{y}}x_{10}\Omega_{r}+\frac{L}{I_{y}}U_{3}\\ x_{10}x_{11}\frac{I_{x}-I_{y}}{I_{z}}+\frac{1}{I_{z}}U_{4} \end{array}\right]. \end{array}$$

**Figure 3:**
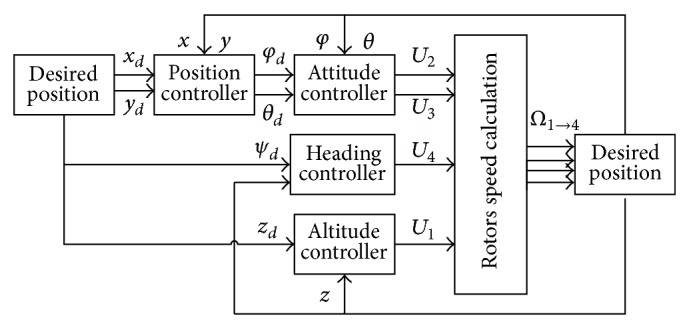
Quadrotor control structure.

It can be seen from (13) that the rotation motions are independent of translational movements and full actuated, while translational motions are underactuated and depend on the rotation motions. Therefore, a control structure with inner and outer loops is designed in which the inner control loop is designed to ensure asymptotic tracking for the desired attitude and altitude and the outer loop is designed to navigate. The structure diagram is shown in Figure 3. As stabilizing attitude is the basis of controlling the quadrotor, quadrotor attitude controller is designed in this paper. In this paper, attitude control strategy of the quadrotor is firstly applying PD controller tuned by Ziegler-Nichols rules to the linear model of quadrotor after feedback linearization and then nonlinear controller consisting of the PD control and feedback linearization item acts on the quadrotor controlled object.

To design attitude controller, the rotation motion equations are separately extracted and simplified, and they are given by

(14) x˙10=a1x11x12−a2x11Ωr+b1U2,x˙11=a3x10x12+a4x10Ωr+b2U3,x˙12=a5x10x11+b3U4,

where *a*_1_ = (*I*_*y*_ − *I*_*z*_)/*I*_*x*_, *a*_2_ = *J*_*r*_/*I*_*x*_, *a*_3_ = (*I*_*z*_ − *I*_*x*_)/*I*_*y*_, *a*_4_ = *J*_*r*_/*I*_*y*_, *a*_5_ = (*I*_*x*_ − *I*_*y*_)/*I*_*z*_, *b*_1_ = *L*/*I*_*x*_, *b*_2_ = *L*/*I*_*y*_, and *b*_3_ = 1/*I*_*z*_.

## 4. Controller Design Principle

### 4.1. Feedback Linearization Theory

The smaller the rotor inertia of quadrotor is, the smaller the impact of gyroscopic effect is. Therefore, during the design of attitude controller, the simplified rotation motion equations are derived by ignoring the influence of the gyroscopic effect item as

(15) x˙10=a1x11x12+b1U2,x˙11=a3x10x12+b2U3,x˙12=a5x10x11+b3U4.

In order to obtain a linear model to make further control easily realize, in this paper, a feedback linearization technique is adopted to linearize a nonlinear system. The control inputs *U*_2_, *U*_3_, and *U*_4_ are chosen as

(16) U2=f2x10,x11,x12+U2#,U3=f3x10,x11,x12+U3#,U4=f4x10,x11,x12+U4#,

where *U*_2_^#^, *U*_3_^#^, and *U*_4_^#^ are new inputs. On this basis, in order to obtain a linear model, the following equations still demand to be met:

(17) a1x11x12+b1f2x10,x11,x12=K2x10,a3x10x12+b2f3x10,x11,x12=K3x11,a5x10x11+b3f4x10,x11,x12=K4x10,

where *K*_2_, *K*_3_, and *K*_4_ are undetermined parameters. According to (17), the feedback linearization items*f*_2_, *f*_3_, and *f*_4_ are given as

(18) f2x10,x11,x12=1b1K2x10−a1x11x12,f3x10,x11,x12=1b2K3x11−a3x10x12,f4x10,x11,x12=1b3K4x10−a5x10x11.

Similarly, if considering gyroscopic effect, feedback linearization items*f*_2_, *f*_3_, and *f*_4_ are derived as

(19) f2x10,x11,x12=1b1K2x10+a2x11Ωr−a1x11x12,f3x10,x11,x12=1b2K3x11−a4x10Ωr−a3x10x12,f4x10,x11,x12=1b3K4x10−a5x10x11.

Substituting (16) into (15), meanwhile considering (17), the linear system can be obtained as

(20) x˙10=K2x10+b1U2#,x˙11=K3x11+b2U3#,x˙12=K4x12+b3U4#.

It can be seen from (20) that the corresponding linear closed-loop system is still stable even considering the gyroscopic effect. In order to prove the stability of the closed-loop system, in this paper, under the condition of *U*_2_^#^ = *U*_3_^#^ = *U*_4_^#^ = 0 and operating points *x*_10_ = *x*_11_ = *x*_12_ = 0, we consider the Lyapunov function

(21) $$\begin{array}{c} V\left(x_{10},x_{11},x_{12}\right)=0.5\left(x_{10}^{2}+x_{11}^{2}+x_{12}^{2}\right). \end{array}$$

Equation (21) is an attitude controller, and it is positive definite near the operating point. The first derivative of the Lyapunov function can be derived using (14) which is a model expression including gyroscopic effect item, (16) and (19); meanwhile, it can be derived using (15) which is a simplified model expression, (16) and (18). It can be seen that the above two first derivatives of the two Lyapunov functions are the same, which denotes the affair that the model including gyroscopic effect item will not affect the stability of the system. The first derivative of Lyapunov function is given by

(22) $$\begin{array}{c} \dot{V}=x_{10}{\dot{x}}_{10}+x_{11}{\dot{x}}_{11}+x_{12}{\dot{x}}_{12}=K_{2}x_{10}^{2}+K_{3}x_{11}^{2}+K_{4}x_{12}^{2}. \end{array}$$

The derivative expressed by (22) is negative definite if *K*_2_ < 0, *K*_3_ < 0, and *K*_4_ < 0, which guarantees that the operating point of the feedback linearization system is asymptotically stable.

Considering ${\dot{x}}_{7}=x_{10}$, ${\dot{x}}_{8}=x_{11}$, and ${\dot{x}}_{9}=x_{12}$, it is obvious that the feedback linearization system, that is, (20), can be described by linear decoupled differential equations of second order; namely,

(23) x¨7=K2x˙7+b1U2#,x¨8=K3x˙8+b2U3#,x¨9=K4x˙9+b3U4#.

The Laplace transformation is applied to (23), which can transform the system from time domain to frequency domain. Further, the open-loop transfer functions of controlled object quadrotor can be obtained as

(24) G1s=X7sU2#s=b1s2−K2s,G2s=X8sU3#s=b2s2−K3s,G3s=X9sU4#s=b3s2−K4s,

where the system is minimum phase system only when *K*_2_ < 0, *K*_3_ < 0, and *K*_4_ < 0.

### 4.2. Ziegler-Nichols PD Parameter Setting Principles

Aiming at the decoupled linear controlled object shown in (23), a simple and practical PD controller is designed. The controller parameters proportion and derivative coefficients are the corresponding proportion and derivative coefficients among the PID parameters adjusted by Ziegler-Nichols PID parameters setting rules. Ziegler-Nichols method is a PID parameter setting method based on stability analysis. The setting of the method for proportion coefficient *K*_*P*_ is, firstly, fixing *K*_*D*_ = *K*_*I*_ = 0; secondly, increasing *K*_*P*_ until the system begins to shock when the closed-loop system poles are on the *jw* axis; finally, multiplying *K*_*P*_ by 0.6. The final *K*_*P*_ is proportion coefficient after setting. Setting formula is given by

(25) KP=0.6Km,KD=KPπ4ωm,KI=KPωmπ,

where *K*_*m*_ is *K*_*P*_ value derived when the system began to shock and *ω*_*m*_ is the frequency of oscillation. *K*_*m*_ and *ω*_*m*_ can be determined using the root locus method. For a given transfer function of the controlled object, one can get its root locus. *K*_*m*_ is the gain traversing the *jw* axis, and the oscillation frequency of the point corresponds to *ω*_*m*_.

### 4.3. Controller Final Form and Parameter Choice

The expressions of linear controllers *U*_2_^#^, *U*_3_^#^, and *U*_4_^#^ can be written after obtaining the PID parameter value. Further, the expressions of nonlinear controllers *U*_2_, *U*_3_, and *U*_4_ also can be written. The actual control loop is discrete time system with sampling period *T*_*s*_. Therefore, in MATLAB simulation, the discrete PID control applies in continuous system [27]. In this paper, a series of sampling points *KT*_*s*_ is selected to replace continuous time *t*, the integral is approximately replaced by rectangle method numerical integral, and the differential is approximately replaced by first-order backward difference; namely,

(26) t≈kT k=0,1,2,…,∫0terrortdt≈T∑j=0kerrorjT=T∑j=0kerrorj,derrortdt≈errorkT−errork−1TT=errork−errork−1T.

The resulting PD controllers *U*_2_^#^, *U*_3_^#^, and *U*_4_^#^ are given by

(27) U2#k=KP2err⁡2k+KD2err⁡2k−err⁡2k−1Ts,U3#k=KP3err⁡3k+KD3err⁡3k−err⁡3k−1Ts,U4#k=KP4err⁡4k+KD4err⁡4k−err⁡4k−1Ts,

where the subscript 2 represents the roll angle loop, the subscript 3 represents the pitch angel loop, the subscript 4 represents yaw angle loop, and *err*⁡2(*k*), *err*⁡3(*k*), and *err*⁡4(*k*) represent the differences between the expected values and the actual values of the roll, pitch, and yaw angles, respectively.

Substituting (18) and (27) into (16) and sorting, nonlinear controllers *U*_2_, *U*_3_, and *U*_4_ are derived as

(28) U2k=1b1K2x10−a1x11x12+KP2err⁡2k +KD2err⁡2k−err⁡2k−1Ts,U3k=1b2K3x11−a3x10x12+KP3err⁡3k +KD3err⁡3k−err⁡3k−1Ts,U4k=1b3K4x12−a5x10x11+KP4err⁡4k +KD4err⁡4k−err⁡4k−1Ts,

where *K*_2_, *K*_3_, and *K*_4_ are the optimal values obtained by using a single optimization method. The goal of optimization process is to stabilize system performance and to make system have faster response and smaller overshoot amount as much as possible. The PID controller parameters are adjusted by Ziegler-Nichols principles.

## 5. Simulation Results

In order to test the control effect of the nonlinear controller proposed in this paper, the simulation experiment was carried out. Single control and joint control for a quadrotor were carried out, respectively. Meanwhile, the control effect comparison was done among the three nonlinear controllers which have the same feedback linearization item and the different linear control parts. The linear part of the controller adopted in this paper is a PD controller with PD parameters tuned by Ziegler-Nichols rules, while the linear parts of the other two nonlinear controllers are, respectively, PID controller with the PID controller parameters tuned by Ziegler-Nichols rules and PD controller with the PD controller parameters tuned by GA. The other two nonlinear controllers are also proposed in this paper and are used to approve the advantages of ZN-PD controller adopted in this paper. Besides, the system robust performance test was done under the condition of noise interference. In this paper, the single control is defined as controlling one of the quadrotor's attitude angles from zero initial state to the desired angle, for example, controlling pitch angle to the desired angle, while the other two attitude angles roll and yaw angles are still located in the initial state; joint control is defined as controlling all of the quadrotor's attitude angles to the expectation, namely, simultaneously controlling roll, pitch, and yaw angles to the desired angles. In this paper, we test robust performance of this system only with the interference of zero-mean white noise. The quadrotor parameters used in this paper are as follows: *m* = 0.4794 kg, *g* = 9.81 m/s^2^, *l* = 0.225 m, *I*_*x*_ = *I*_*y*_ = 0.0086 kg·m^2^, *I*_*z*_ = 0.0172 kg·m^2^, *J*_rotor_ = 3.7404 × 10^−5^, *b* = 3.13 × 10^−5^, *d* = 9 × 10^−7^, *K*_2_ = −14.6211, *K*_3_ = −14.6211, *K*_4_ = −32, and *U*_1_ = *m*∗*g*.

**Figure 4:**
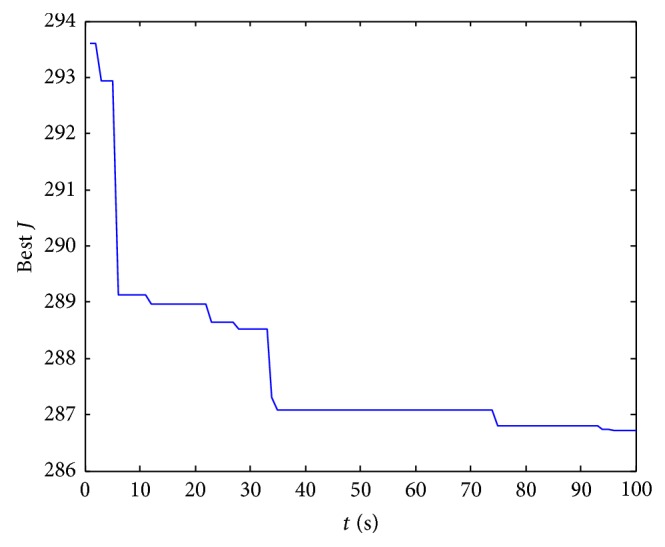
The target value optimization diagram using GA to tune PID parameter values of roll angle loop.

The transfer function for the roll angle loop after feedback linearization is *G*_1_(*s*) = *X*_7_(*s*)/*U*_2_^#^(*s*) = *b*_1_/(*s* ^2^ − *K*_2_ *s*) = 26.1628/(*s* ^2^ + 14.6211*s*). The target value optimization diagram using GA to tune PID parameter values of this attitude loop is shown in Figure 4. The ultimate values and initial values scope of PID parameters in GA parameter optimization are shown in Table 1.

The PID parameter modulation processes for pitch angle and yaw angle loops are similar to the modulation process for roll angle loop. Just different initial values of PID parameters are applied to pitch angle and yaw angle loops. The PID parameter values tuned by Ziegler-Nichols rules and GA are shown in Table 2. The positive and negative 20 percent of PID parameters for each attitude loop tuned by Ziegler-Nichols rules are chosen as the initial values of PID parameters for GA optimization.

**Figure 5:**
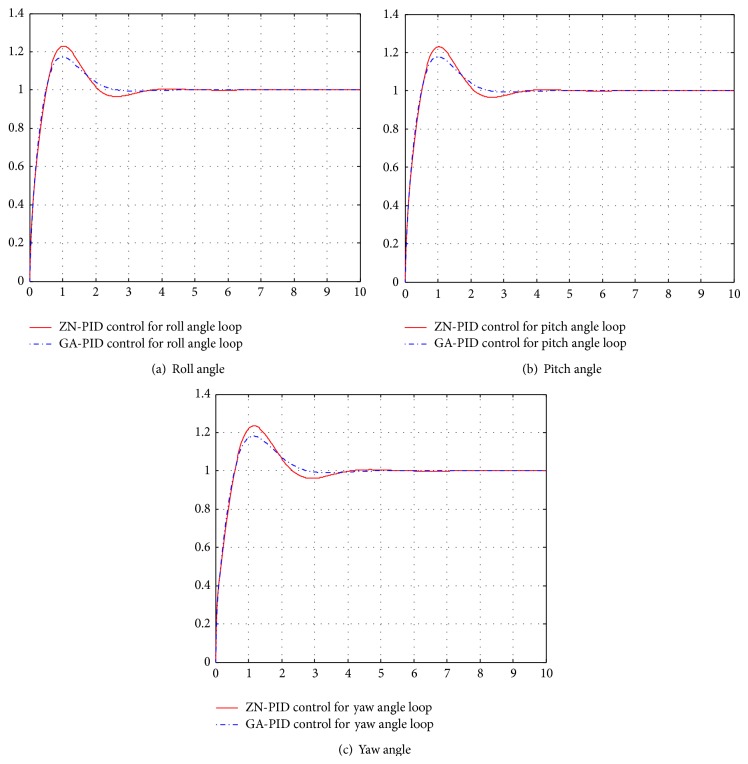
The control effects compare diagrams between ZN-PID and GA-PID for three attitude angles. (a) The control effects compare diagram of the roll angle loop. (b) The control effects compare diagram of the pitch angle loop. (c) The control effects compare diagram of the yaw angle loop.

The control effects compare diagrams between ZN-PID (PID control with PID parameters tuned by Ziegler-Nichols rules) and GA-PID (PID control with PID parameters tuned by GA) for three attitude angle loops which are shown in Figure 5. It can be seen from Figure 5, for three attitude angle loops, the control effects of GA-PID are all better than those of ZN-PID.

The following ZN-PD control (PD control with PD parameters tuned by Ziegler-Nichols rules), GA-PD control (PD control with PD parameters tuned by GA), and ZN-PID control (PID control with PID parameters tuned by Ziegler-Nichols rules), respectively, stand for the linear control parts of three attitude controllers appearing in this paper. GA-PD control and ZN-PID control are used to compare control effect with ZN-PD control adopted in this paper. The nonlinear parts of three attitude controllers are the same. For convenience, the names of the linear control parts of three different kinds of nonlinear controllers, respectively, represent the corresponding nonlinear controllers.

**Figure 6:**
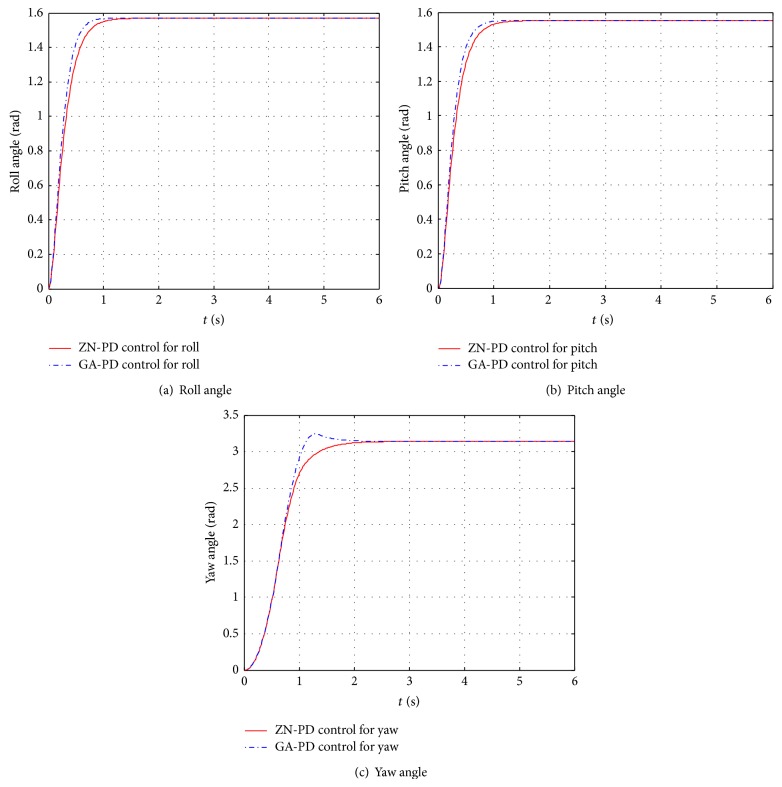
The control effects compare diagrams between ZN-PD and GA-PD of separately controlling an attitude angle to the desired angle, while the rest attitude angles remain 0. (a) The control effects compare diagram of the roll angle. (b) The control effects compare diagram of the pitch angle. (c) The control effects compare diagram of the yaw angle.

The control response comparison diagrams of the single ZN-PD control and the single GA-PD are shown in Figure 6. It can be seen from Figure 6 that ZN-PD control and GA-PD control can control three attitude angles to any value in their domain of definition; for the roll and pitch angle loops, the control effects of GA-PD are better than the one of ZN-PD; for the yaw angle loop, GA-PD control has a large overshoot while ZN-PD quickly and without overshoot reaches the desired value.

**Figure 7:**
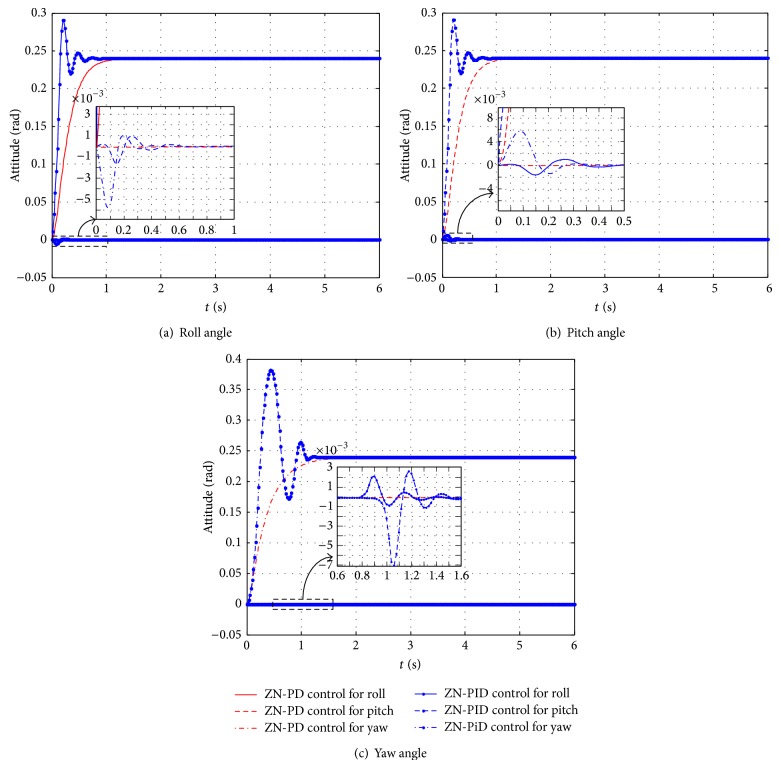
The control effects compare diagrams between ZN-PD and ZN-PID of separately controlling an attitude angle to the desired angle, while the rest attitude angles remain 0. (a) The control effects compare diagram of the roll angle. (b) The control effects compare diagram of the pitch angle. (c) The control effects compare diagram of the yaw angle.

For the quadrotor controlled object, the larger overshoot is fatal since it often leads to instability. The domains of definition of three attitude angles *ϕ*, *θ*, and *ψ* are −*π*/2 < *ϕ* < *π*/2, −*π*/2 < *θ* < *π*/2, and −*π* < *ψ* < *π*, respectively. Simulation experiments demonstrate that ZN-PID control can only control every attitude angle to 0.24 rad. The ZN-PD control and the ZN-PID control effect comparison is done when separately controlling an attitude angle to the desired angle, while the rest attitude angles remain 0. The control effect comparison diagrams are shown in Figure 7. It can be seen from the Figure 7 that ZN-PD control is apparently superior to the ZN-PID control. ZN-PD control can quickly and without overshoot control an attitude angle to its expectation and interactive responses among three attitude angles under the ZN-PD control are smaller than those under the ZN-PID control.

**Figure 8:**
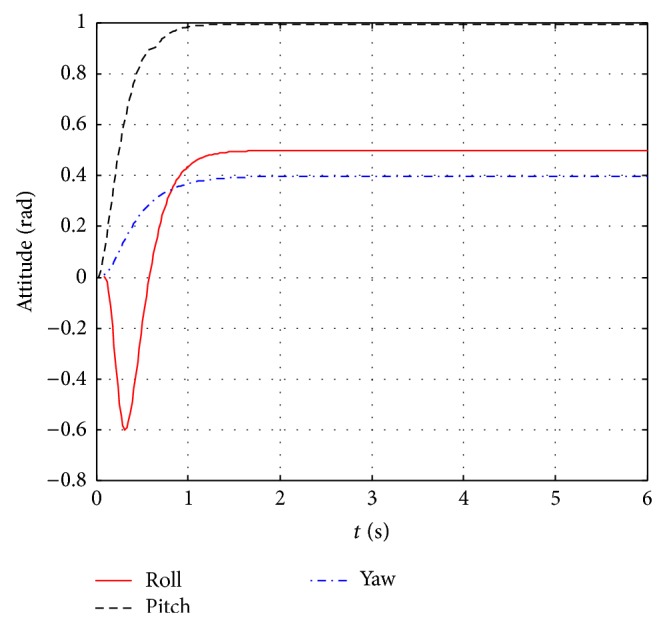
ZN-PD control responses of jointly controlling three attitude angles to the expectations.

**Figure 9:**
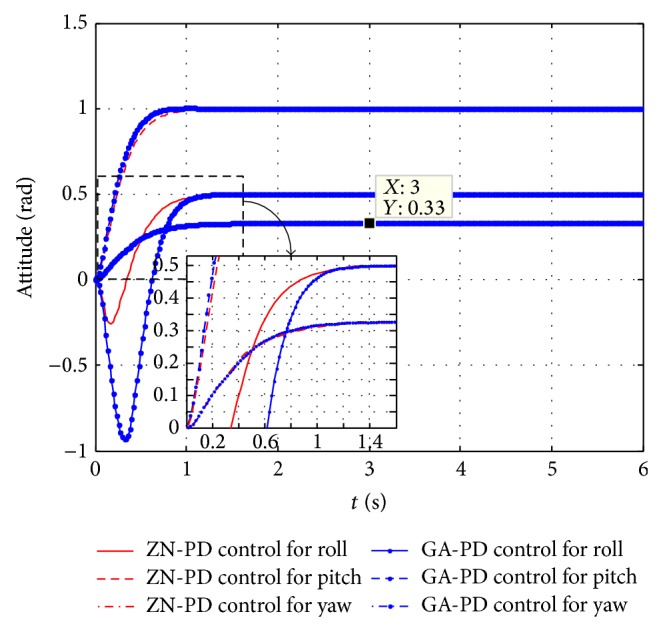
The control effects compare diagram between ZN-PD and GA-PD of jointly controlling three attitude angles to the expectations.

The further control effects compare between ZN-PD control and GA-PD control and can be seen from Figures 8 and 9. The control response curves jointly control three attitude angles to their expectations and are shown in Figure 8. It can be seen from Figure 8 that the ZN-PD controller can jointly control three attitude angles to arbitrary angle within the scope of (0.5,1,0.4) (rad), while simulation experiments demonstrate that GA-PD control can only jointly control three attitude angles to arbitrary angle within the scope of (0.5,1,0.33) (rad). The control effect comparison diagrams between ZN-PD and GA-PD of jointly controlling roll angle, pitch angle, and yaw angle to the expectations 0.5 rad, 1 rad, and 0.33 rad are shown in Figure 9. It can be seen from Figure 9 that ZN-PD control can make attitude angles quickly reach to the desired values and has smaller negative overshoot than GA-PD control in roll angle loop. It is obvious that the control effect of ZN-PD is better than one of GA-PD in jointly controlling three angles to the expectations.

**Figure 10:**
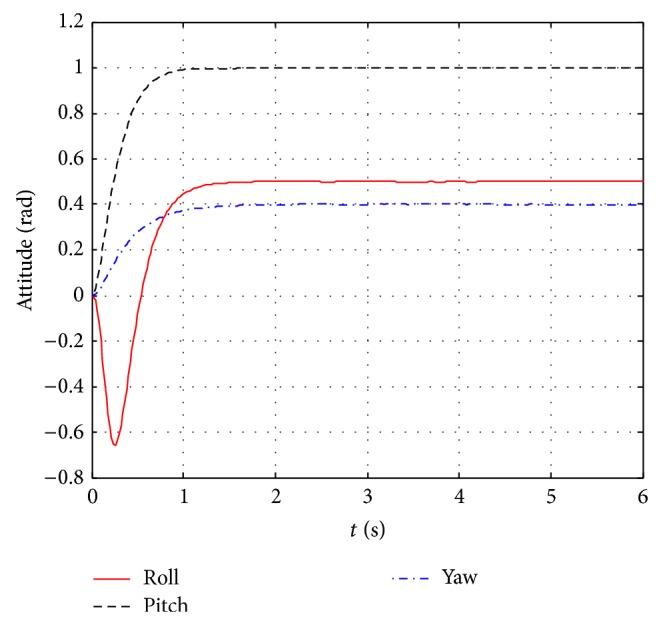
ZN-PD control responses with noise interference of jointly controlling three attitude angles to the expectations.

It can be seen by synthesizing Figures 6, 8, and 9 that the control effect of ZN-PD is superior to the one of GA-PD. It can be seen by synthesizing Figures 6, 7, 8, and 9 that the control effect of ZN-PD is superior to the one of ZN-PID and GA-PD. In a word, the attitude controller adopted by this paper is simple and has very good control effect. The robustness tests for the ZN-PD control are shown in Figures 8 and 10. Figure 8 corresponds to the joint ZN-PD control response curve without noise interference.  Figure 10 corresponds to the joint ZN-PD control response curve with noise interference. It can be seem from Figures 8 and 10 that the controller design method proposed in this paper is highly robust.

The robustness of ZN-PD controller results from the controller constituent. From the perspective of the frequency domain, in PID control, integral acts on low frequency band of the system to improve the stability of the system, and differential acts on middle frequency band of the system to improve the dynamic performance of system. But, in quadrotor control, the ZN-PD control effect is better than the ZN-PID control effect; the reason is that the integrator characteristics contradict the characteristics of quadrotor controlled object. The essence of increasing the integrator is adding a pole, which can eliminate the steady-state error of the system. But strong integral action will increase the amount of overshoot so as to make the system unstable. Nevertheless, quadrotor is a controlled object with narrow domain of definition, and the domains of definition of three attitude angles *ϕ*, *θ*, and *ψ* are −*π*/2 < *ϕ* < *π*/2, −*π*/2 < *θ* < *π*/2, and −*π* < *ψ* < *π*, respectively. Therefore, when integrator acts on the quadrotor controlled object, quadrotor will become unstable as long as producing certain amount of overshoot, while this amount of overshoot will not affect the stability of the controlled object with wide domain of definition. When the noise interference is added to the quadrotor, the larger overshoot will appear in ZN-PID control, but the large overshoot will not appear because the integral action is not included and differential control can reduce overshoot and speed up the response in ZN-PD control.

The GA-PD control is an intelligent control method; it should have better control effect than ZN-PD control. But it can be seen from the simulation experiment proceeded in this paper that, in single control, for the roll and pitch angle loops, the control effects of GA-PD are better than those of ZN-PD, but, for the yaw angle loop, GA-PD control has a large overshoot while ZN-PD can quickly and without overshoot reach the desired value. For the quadrotor controlled object, the larger overshoot is fatal, which can be seen from the joint control. GA-PD control can only jointly control three attitude angles to arbitrary angle within the scope of (0.5,1, 0.33) (rad) which is smaller than (0.5,1, 0.4) (rad) of ZN-PD. the smaller the maximum angle is, the more the chance of unstable in afterward position control is. Meanwhile, the control effect of GA-PD is worse than the one of ZN-PD in jointly controlling three attitudes to the small values between GA-PD and ZN-PD. Moreover, the GA-PD control costs more system resources and more adjusting time, and further has the worst real-time which is more than the ZN-PD control. Therefore, the ZN-PD controller is selected in this paper.

In one word, the control effect of ZN-PD control proposed in this paper is superior to the control effects of ZN-PID and GA-PD. ZN-PD control is a simple and practical method.

## 6. Conclusion

The attitude controller adopted by this paper is a nonlinear controller. It consists of a linear control part and a nonlinear control part, where the linear control part is a PD controller which parameters were tuned by Ziegler-Nichols rules, and the nonlinear control part is a feedback linearization item which converts a nonlinear system into a linear system. The control effect of the attitude controller adopted by this paper is better than the control effects of the other two nonlinear controllers. The nonlinear parts of the other two nonlinear controllers are the same as the nonlinear part of the attitude controller proposed in this paper. The linear parts are, respectively, PID controller with the PID controller parameters tuned by Ziegler-Nichols rules and PD controller with the PD controller parameters tuned by GA. Besides, the attitude controller adopted by this paper is highly robust and the controller design method is a simple and practical one in engineering. The controller design ideas can also be used to other nonlinear controlled objects.

## Figures and Tables

**Table 1:** The ultimate values and initial values scope of PID parameters in GA parameter optimization.

	*P*	*I*	*D*
The ultimate PID parameter values	2.2310	2.9996	0.1969
The initial value scopes of PID parameters	[1.49344, 2.24016]	[2.97896, 4.46844]	[0.1872, 0.2808]

**Table 2:** PID parameter values tuned by Ziegler-Nichols rules and GA.

Attitude	PID value					
	PID parameter values tuned by Ziegler-Nichols rules			PID parameter values tuned by GA		
	*P*	*I*	*D*	*P*	*I*	*D*
Roll angle	1.8668	3.7237	0.2340	2.2310	2.9996	0.1969
Pitch angle	1.8572	3.6941	0.2334	2.1756	3.0239	0.2022
Yaw angle	1.5064	3.0067	0.1887	1.8013	2.479	0.1528
